# How do carbon emissions trading impact the financialization of non-financial companies? Evidence from a quasi-natural experiment in China

**DOI:** 10.1371/journal.pone.0296277

**Published:** 2023-12-27

**Authors:** Wenhao Ma, Xuwen Yan

**Affiliations:** School of Finance, Zhongnan University of Economics and Law, Wuhan, Hubei, China; University of Murcia: Universidad de Murcia, SPAIN

## Abstract

This study examines whether and how carbon trading policy impacts the financialization of non-financial firms, using China emission trading scheme as a quasi-natural experiment. We find that the carbon trading policy exerts a substantial and enduring inhibitory effect on corporate financialization. Our findings are robust to possible result bias and more precise control group. Additionally, we explore potential channels through which carbon trading policy can affect financialization, and find that it curbs financialization by reducing financing constraints. Finally, we demonstrate that the relationship between carbon trading policy and financialization of non-financial companies is moderated by company’s ownership, region, and industry competition.

## 1. Introduction

Threats over global warming linked to carbon dioxide (CO_2_) emissions from human activity have recently become salient [1], which are received growing attention by academics, media, and politicians [2]. Countries are actively adopting carbon reduction action and may introduce significant limits on CO_2_ emissions within the next decade to accomplish the 2°C or even 1.5°C target set in the Paris climate agreement [1, 3]. Previous research has documented that the carbon emissions trading has significant effects on firm-level outcomes [4–7] (e.g. innovation, performance, and stock returns). However, the literature widely neglects its influence on corporate financialization.

This study aims to explore how carbon emissions trading affects the financialization of non-financial companies (NFC). Corporate financialization can be defined as NFC increasing investment in financial assets while reducing productive investment [8]. The financialization of NFC has become a common phenomenon in emerging markets. Taking China as an example, the disproportionately high growth beyond real economic needs of China’s financial sector has enabled financial assets to bring substantial profits to enterprises [9], which exacerbates the profitability gap between the financial sector and the non-financial sector. As a result, NFC are forced to invest in more profitable financial assets due to the downturn in entities. However, existing research has shown that financialization makes the surplus capital of NFC increasingly used for speculation and arbitrage instead of their main businesses [8], such as innovation or production improvement, thereby reducing the company’s core business potential and future profitability [10, 11]. This may cause a vicious circle of “low profit ‐ financialization ‐ lower future profitability” for NFC. The case of Shanghai RAAS, whose high level of financialization led to stock price crash and market capitalization loss of over ten billion yuan, shows that the harm of financialization of NFCs have been revealed in emerging markets.

However, the possible impact of carbon emissions trading on corporate financialization is inconclusive. As a typical environmental regulation, carbon emissions trading can not only further damage NFC’s profits through compliance costs, but also reduce the return on entities’ investments, thereby weakening investor confidence in polluting companies, leading to underperformance of company stocks [12, 13]. In this case, the unintended consequences of carbon emissions trading will undermine its policy effectiveness by exacerbating the financialization. But the emissions trading may also reduce the financialization of NFCs by increasing the direct and indirect profits of them. Therefore, it is necessary to assess the relationship between carbon emissions trading and the financialization of NFC.

To conduct the examinations, we use China as a laboratory since it provides an ideal research context. First, as the world’s largest carbon emitters and an emerging economy, China approved carbon emissions trading pilots in 7 provinces and cities since 2011 to promote the low-carbon economy transition, all of which launched trading in 2014. The implementation of carbon emissions trading pilots can be seen as a quasi-natural experiment for identifying the causal relationships between carbon emissions trading and financialization, with strictly exogenous characteristic that prevent the possibly reverse shaping of carbon emissions trading by financialization. Second, the financialization of NFC and low-carbon development is a prominent social issue in China. China not only needs low-carbon economic transformation, but also rapid economic development. However, if carbon policies intensify the financialization of NFC, it will have a negative impact on the real economy in the future. Hence, studying the impact of carbon emissions trading on NFC financialization based on China context has practical significance.

Based on the differences between covered companies and non-covered ones before and after the carbon emissions trading pilots, we construct a difference-in-differences (DID) model and link it with the financialization index [11, 14] to explore the impact of carbon emissions trading on the financialization of NFC. Using a sample of China listed NFC over the period of 2008 to 2020, we find that the financialization degree of NFC located in pilot areas significantly decreases. This result still holds when we validate the robustness of our research. Hence, carbon emissions trading effectively inhibits the financialization of NFC.

Then, we explore the influence channel of carbon emissions trading. We find that carbon emissions trading can inhibit corporate financialization by alleviating corporate financing constraints. One possible reason for this result is that companies can gain direct economic benefits by selling carbon emissions rights. Li et al. [15] also pointed out that carbon trading can improve enterprises’ transparency of carbon information, which can reduce information asymmetry and create an environmentally friendly corporate image, thereby bring indirect profits to the firms.

Finally, we conduct several cross-sectional tests in terms of company ownership, company location, and the degree of industry competition. We find that non-state-owned ownership, eastern location, and a high level of industry competition promote the inhibitory effect of carbon emissions trading on financialization. Non-state-owned enterprises turn to holding more financial assets to survive in the context of the downturn in the entities’ economy leading them to be more significantly affected by carbon trading policies. Whereas firms in eastern regions and highly competitive industries have to hold more financial assets under the combined pressure of competition and shrinking markets, which makes them very sensitive to the alleviation of financing constraints brought by carbon emissions trading.

Our research makes several contributions to the existing literature. First, to our best knowledge, we are the first to explore the relationship between carbon emissions trading and the financialization of non-financial companies. As one of the effective means to curb climate change, carbon emissions trading has attracted the attention of all countries. Previous research has proved carbon emissions trading has real effects on firms [4–7], but neglected its influence on corporate financialization. Our study extends the consequences of carbon emissions trading at the company level. Second, we take China emission trading scheme pilots as a quasi-natural experiment, which captures the main aspect of carbon policies and helps us to identify the causality between carbon trading policies and corporate financialization using a difference-in-differences model. In this context, our paper also relates to literature on the economic consequence of environmental regulation [16–18]. Third, we explore the mechanism through which carbon emissions trading inhibits corporate financialization. We contribute to the literature by directly identifying corporate financing constraints as the influence channel of carbon emissions trading. Finally, our results provide clear policy implications for low-carbon transitions in emerging markets. Nowadays, more than half of the world’s top 10 carbon dioxide emitters are emerging and developing countries [19]. For them, non-financial firms are not only the main carbon emissions source, but also the key driver of economic development. Therefore, it is important to assess the impact of carbon emissions trading on the financialization of non-financial companies in emerging markets, because financialization is one of the obstacles to the corporate development. Taking the typical emerging market China as the background, we find that carbon emissions trading can reduce the financialization of non-financial companies, thereby promoting the development of a low-carbon economy, which can serve as a reference for other emerging markets.

The rest of this paper is organized as follows. Section 2 discusses the institutional background and hypothesis development. Section 3 introduces the sample construction and research design. Section 4 reports the empirical results, and Section 5 presents the influence channels of carbon trading policies. Then, section 6 presents the further tests conducted and Section 7 concludes.

## 2. Institutional background and hypothesis development

### 2.1 Institutional background

Green and low-carbon development has become a shared objective within the international community [20]. In pursuit of this, China has embarked on an eco-friendly transformation of its economy over the past few decades, transitioning from rapid economic growth to a focus on high-quality economic development [21]. Compared with developed countries, China’s decarbonization policy started relatively late. During the “11th Five-Year Plan”, China began to incorporate energy conservation and emission reduction into the national economic and social development plan. 2009 can be marked as the first year of carbon reduction in China. At the United Nations Climate Change Conference in Copenhagen in 2009, China announced that it will reduce carbon intensity by 40% to 45% in 2020 compared with 2005, which marks that reducing carbon emissions has been officially incorporated into China’s development strategy. At the beginning of the “12th Five-Year Plan”, carbon emission intensity control was incorporated into the national economic and social development plan and became one of the main binding indicators. The “14th Five-Year Plan” outline further clarifies the carbon reduction policy. The plan requires that China’s carbon dioxide emissions per unit of GDP must be reduced by 18% within 5 years. Afterwards, the Chinese government proposed the goal of reaching a carbon peak by 2030 and carbon neutrality by 2060.

To achieve the carbon reduction goal in the development strategies, the Chinese government has implemented several measures. Among these, one of the most impactful strategies is the adoption of carbon emissions trading pilots [22]. In 2011, China’s National Development and Reform Commission (NDRC) released *the Work Plan for Controlling Greenhouse Gas Emissions During the 12th Five-Year Plan Period*. This plan allocated specific targets for reducing carbon intensity to all provinces, autonomous regions and centrally-administered municipalities in China. The plan’s objective was to establish exemplary low-carbon technologies, products, and communities, thereby significantly enhancing China’s capability to manage greenhouse gas emissions. According to the plan, China aimed to notably decrease carbon emissions per unit of gross domestic product (GDP) and achieve a 17% reduction in national carbon emissions per unit of GDP by 2015, as compared to the baseline of 2010.

**Fig 1:**
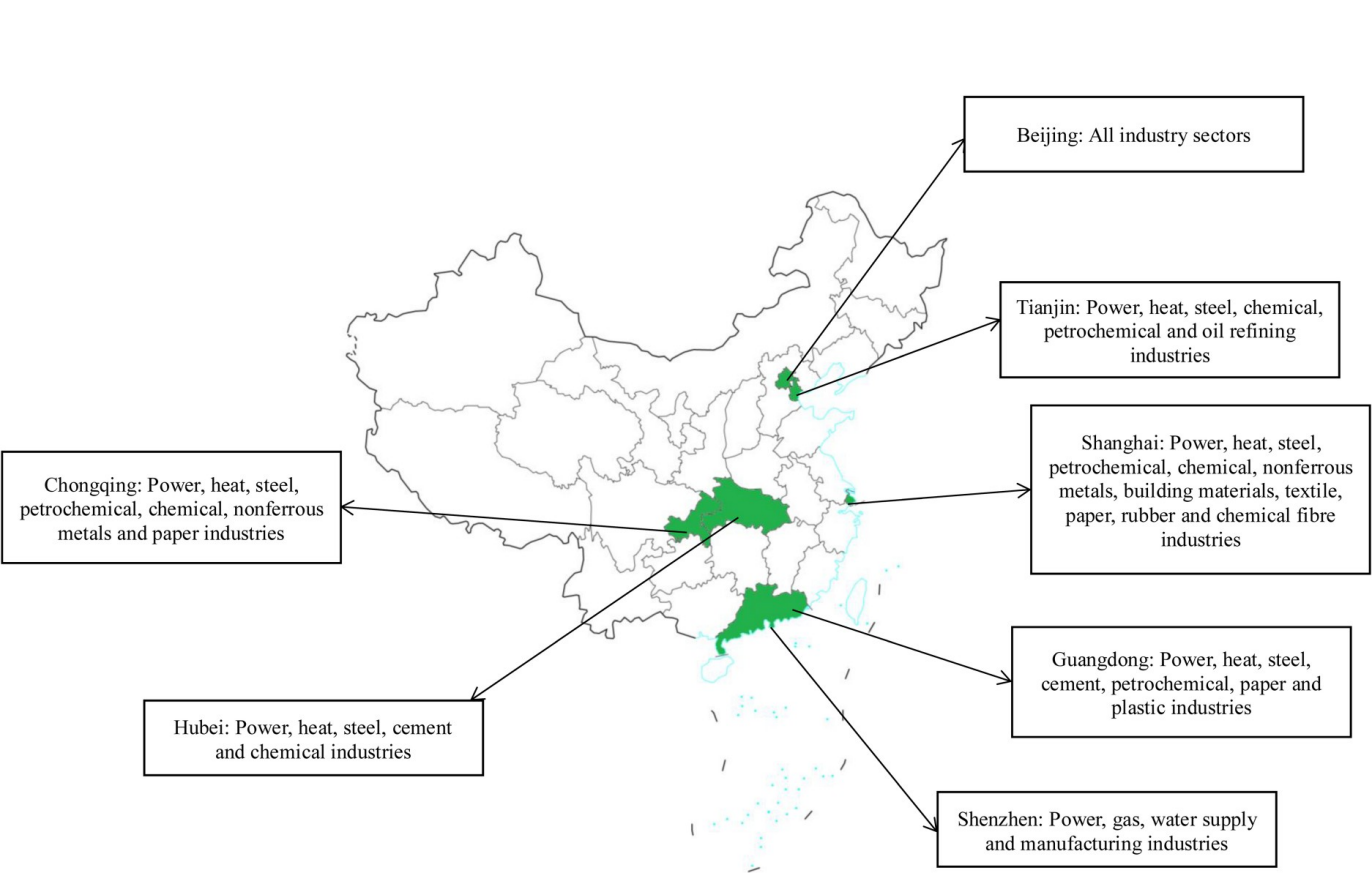
The pilot areas and covered industries. Fig 1 shows China emission trading scheme (ETS) pilots in 7 provinces and cities with industries covered by each pilot.

The plan designated Beijing, Shanghai, Tianjin, Hubei, Guangdong, Shenzhen, and Chongqing as the first batch of pilot areas for the emissions trading. These pilot areas encompass the eastern coastal region as well as the central and western regions. By 2014, all carbon trading markets in these seven pilot provinces and cities had been established, covering more than 20 industries such as steel, electricity, cement, etc. The specific pilot areas and the industries they encompass are shown in Fig 1. From 2013 to 2019, the total amount of carbon trading within the pilot market increased from 445,500 tons to 31 million tons with the transaction volume raising from RMB 25 million to RMB 952 million. These developments have led to significant economic impacts [6].

Given that the characteristics and behaviors of firms cannot influence the formulation and promulgation of carbon emissions trading pilots, these pilots can be regarded as a strictly exogenous quasi-natural experiment. As a result, they provide an ideal context devoid of endogeneity for examining the impact of carbon trading on the financialization of NFC. This is due to the post-policy differences among companies resulting from an external shock. Specifically, the carbon emissions trading pilots introduce two significant differences: firstly, carbon emission allowances can be traded after the pilots, and secondly, carbon trading is restricted to covered industries within pilot areas. Consequently, based on the two-dimensional changes brought about by the pilots, we construct a difference-in-differences model to identify the causal influence of carbon trading on the financialization of NFC.

### 2.2 Hypothesis development

Considering that environmental regulations have the capacity to shape corporate behavior, carbon trading policies can indeed impact corporate financial decisions [23]. Previous research has pointed out that non-financial companies are primarily driven to hold financial assets due to liquidity reserves and compensation for operating losses [24, 25]. Consequently, we aim to analyze the potential impact of carbon trading policies through these two motivations.

#### 2.2.1 Liquidity reserves motivation

The reservoir theory believes that companies hold financial assets to mitigate uncertainties in future cash flows [26, 27]. However, environmental regulations can increase the unpredictability of expected cash flow of business entities.

On the one hand, the compliance costs caused by environmental regulations, such as emission fees and expenses for equipment upgrades, can directly erode corporate profits, thereby reducing cash flow [28]. On the other hand, financial institutions may choose to reduce the scale of loans or increase borrowing costs for enterprises, especially those heavily engaged in pollution, in order to avoid potential credit risks arising from environmental issues under stringent environmental regulations, which makes loan financing more difficult [29]. Concurrently, the policy risks tied to environmental regulations can undermine investors’ confidence in polluting companies as these risks permeate the capital market. This could result in a diminished performance of company stock, subsequently increasing the difficulty of corporate equity financing [13].

Furthermore, the presence of information asymmetry along with the inherently exogenous nature of environmental regulations restricts firms to predicting future shifts in these regulations solely based on their present intensity. During this phase, the increasingly stringent environmental regulations may lead to increased volatility in companies’ expected cash flow. As a result, firms may opt to augment their investment in financial assets to alleviate potential future financing constraints and survival risks. This, in turn, prompts non-financial companies to elevate their holdings of financial assets as a means to maintain liquidity in response to environmental regulations.

#### 2.2.2 Losses compensation motivation

Neoclassical economics asserts that environmental policies can mitigate environmental pollution by internalizing negative externality costs [30]. However, these additional cost increments caused by environmental regulations usually have to be borne by companies themselves, rather than being offset by state subsidies [9, 31]. Consequently, companies are compelled to allocate their revenues towards reducing pollution emissions, a move that can potentially impact profits adversely [12, 32]. Based on the principal-agent theory, managers often prioritize short-term gains over risk considerations, particularly when confronted with earnings pressure [33]. Therefore, financial assets offering high returns and high risks might emerge as a lifeline for managers, serving to offset the burden of compliance costs associated with environmental regulations.

On the basis of the above analysis, we formulate the following hypothesis.

Hypothesis: Carbon trading policy intensifies the degree of financialization of Chinese non-financial companies.

## 3. Data and variables

### 3.1 Data and sample selection

In order to evaluate how carbon trading policy impacts the financialization of non-financial companies, we collect firm data from China Research Data Services Platform (CNRDS) and China Security Market and Accounting Research (CSMAR).

This study takes all listed enterprises in China from 2008, which is the sixth year before ETS implementation, to 2020 as the initial sample. This time frame is chosen since the available post-ETS firm-year data ends in 2020, which is also the sixth year following the ETS promulgation. The selection process adheres to the subsequent criteria. First, we exclude financial, insurance and real estate listed companies. Second, we eliminate the items with missing data. Third, we exclude the enterprises (marked as ST or *ST) that suffered serious losses in the sample period according to Tian et al. [34]. Moreover, we winsorize continuous variables at the 1% and 99% percentiles to control the effect of extreme value. After data processing, the final sample contains 28,600 observations.

### 3.2 Measuring corporate financialization

Following the views of Xu and Xuan [11] and Akkemik and Özen [14], we use the ratio of financial assets to total assets to measure the financialization of enterprises. This ratio can present the inclination of firms’ investment choices, that is, the willingness to financialize. The specific indicators are constructed as follows: the sum of transactional financial assets, derivative financial assets, the amount of loans and advances issued, the net amount of financial assets available for sale, net investments held to maturity, and net investment real estate divided by the year-end total assets. Given the rapid expansion of China’s real estate at this stage, the real estate investment of many firms has transitioned from the self-use purpose to profit-seeking, which is consistent with the definition of financial assets in this paper. Hence, we include net real estate investment in financial assets. However, cash is excluded from the definition of financial assets since companies predominantly maintain currency funds for their needs of daily production and operation. These funds generally cannot yield capital appreciation to the company [35].

### 3.3 Research design

We employ a difference-in-differences (DID) model to identify the impact of carbon trading policy on corporate financialization. The basic logic of the DID model is to observe the treatment group and the control group before and after the policy shock. Changes in external policies only affect the treatment group, which leads to the difference between the treatment group and the control group after the policy shock. This difference is the result of external influences. Therefore, the two-dimensional changes of ETS pilot on intercompany (belonging to the affected industry or not) and time (before and after the implementation) can be used as a laboratory to identify the causal impact of carbon trading policy on financialization through DID model.

The DID model has several advantages. First, the DID model compares the differences between the treatment group and the control group under exogenous shocks to exclude non-policy-related influence. Consequently, it isolates the net effect of the policy and thus reduce endogeneity [36]. Second, Tang et al. [37] stated that the results of the DID model are more reliable than traditional methods that use dummy variables to evaluate policy effects. Finally, it is widely used in causality identification of carbon trading policy and company reactions in context of China [4, 6, 38]. Hence, we build the following model (1):

$$Fin_{i,t}=\beta_{0}+\beta_{1}Treat_{i}*Post_{t}+\beta_{2}Controls_{i,t}+\mu_{j}+\gamma_{c}+\theta_{t}+\varepsilon_{i,t,j,c}$$ (1)

where the subscript i and t means company i and year t, respectively. Fin_i,t_ represents degree of corporate financialization, which is measured by the ratio of financial assets to total assets, of company i in year t. A higher Fin_i,t_ represents that companies are more inclined to invest in financial assets. We connect the enterprises in ETS pilot area, covering industries with the year of all pilot carbon markets starting transactions, to construct Treat_i_ * Post_t_ as an explanatory variable according to Qi et al. [6] and Zhang and Duan [39]. For Treat_i_, it equals 1 if the firm is in the industries actually covered by the ETS pilots area, and 0 otherwise. Post_t_ equals 1 if the year is greater than or equal to 2014, which is the starting year of trading in all ETS pilot, and 0 otherwise.

We also include several widely used control variables in the model based on Duchin [40] and Feng et al. [41], which are related to financialization. These variables include Lev (a proxy for enterprise leverage), LnSize (a control for firm size), SS and Rid (controls for corporate governance), LnAge (a proxy for enterprise maturity), ROA (a control for company performance), and Growth (a control for company growth ability). The specific definitions of these variables are detailed in the S1 Table.

Furthermore, according to Chen et al. [4] and Qi et al. [6], we add θ_t_ (year fixed effects) and μ_j_ (industry fixed effect) to control the potential impact of time-varying economic cycles and the unobserved time-invariant characteristics across industries on financialization. Additionally, to further account for unobservable time-invariant differences across cities that may affect firms’ financial assets holding, we refer to Liu et al. [38] and add γ_c_ (cities fixed effects). The main coefficient of interest in model (1) is β_1_. A positive coefficient of β_1_ would indicate the carbon trading policy intensifies the degree of financialization of Chinese non-financial companies, vice versa.

### 3.4 Descriptive statistics

Table 1 shows the descriptive statistics of the main variables. The minimum value of Fin is 0.000, and the maximum value is 0.981, indicating significant differences in the degree of financialization of Chinese non-financial companies. The mean and standard deviation of Fin are 0.036 and 0.080, respectively, which are consistent with the existing literature [41], thus confirming the absence of any deviations in the sample selection. The minimal data set of these variables are detailed in the S1 Data.

**Table 1:** Descriptive statistics.

Variable	Mean	Std dev	Min	Q1	Median	Q3	Max	N
**Fin**	0.036	0.080	0.000	0.000	0.006	0.034	0.981	28,600
**Treat**	0.120	0.325	0.000	0.000	0.000	0.000	1.000	28,600
**Post**	0.661	0.473	0.000	0.000	1.000	1.000	1.000	28,600
**ROA**	0.036	0.069	-0.316	0.013	0.037	0.067	0.206	28,600
**LnSize**	22.053	1.254	19.482	21.154	21.900	22.782	25.805	28,600
**Lev**	0.427	0.207	0.053	0.265	0.419	0.578	0.978	28,600
**LnAge**	2.754	0.386	1.386	2.565	2.833	3.045	3.434	28,600
**SS**	4.462	2.145	2.000	3.000	4.000	5.000	15.000	28,600
**Rid**	0.388	0.100	0.000	0.333	0.375	0.444	0.667	28,600
**Growth**	0.172	0.446	-0.597	-0.029	0.103	0.262	2.967	28,600

The mean values of the control variables are as follows: ROA is 0.036, LnSize is 22.053, Lev is 0.427, LnAge is 2.754, SS is 4.462, Rid is 0.388, and Growth is 0.172. Overall, the control variables fall within a reasonable range, as they align with findings from existing research [41, 42].

## 4. Carbon trading policy and corporate financialization

### 4.1 Baseline regression results

We employ the OLS estimation to model (1) and explore the impact of carbon trading policy on the financialization of non-financial firms. Table 2 presents the regression results. Column (2) adds control variables to column (1).

**Table 2:** Pilot carbon trading policy and corporate financialization.

	(1)	(2)
Variable	Fin	Fin
Treat * Post	-0.006***	-0.005**
	(0.002)	(0.002)
**ROA**		-0.003
		(0.007)
**LnSize**		-0.001**
		(0.000)
**Lev**		-0.042***
		(0.003)
**LnAge**		0.024***
		(0.001)
**SS**		-0.001**
		(0.000)
**Rid**		0.003
		(0.005)
**Growth**		-0.001
		(0.001)
**Year fixed effect**	Yes	Yes
**Industry fixed effect**	Yes	Yes
**City fixed effect**	Yes	Yes
**Adj.R** ^**2**^	0.138	0.154
**Observations**	28,600	28,600

Note. This table shows the impact of pilot carbon trading policy on corporate financialization. Column (2) adds control variables to the column (1). Fin represents degree of corporate financialization, where a higher value indicates that companies are more inclined to invest in financial assets. Treat is a dummy variable, which equals 1 if the firm is in the industries actually covered by the ETS pilots area, and 0 otherwise. Post is a dummy variable that equals 1 if the year is greater than or equal to 2014, which is the starting year of trading in all ETS pilots, and 0 otherwise. Control variables are as follows: (1) ROA is the return on assets, which equals to net profits divided by total assets. (2) LnSize represents the natural logarithm of total assets. (3) Lev represents the ratio of total debt to total assets. (4) LnAge represents the natural logarithm of company age. (5) SS is the number of the supervisory board. (6) Rid represents the ratio of the number of independent directors to the total number of board directors. (7) Growth represents the growth rate of the company’s main business. Cluster-robust standard errors are in parentheses. *, **, and *** indicate significance at 10%, 5%, and 1% levels, respectively.

In column (1), the coefficient of Treat * Post is significantly negative at the 1% level, indicating that the carbon trading policy effectively prevents the intensification of corporate financialization. After the inclusion of control variables, the coefficient of Treat * Post is still significantly negative.

Surprisingly, the regression results are contrary to our hypothesis that carbon trading policy curbs the financialization of non-financial firms. More specifically, the carbon trading policy with compliance cost does not lead managers to increase investments in financial products for liquidity reserving and losses compensation. On the contrary, companies in the pilot covered industries cut down their financial assets investment after the launch of carbon trading.

Regarding the control variables, both LnSize and Lev are negatively related to Fin, indicating that large company scale and adequate financing can reduce enterprises’ willingness of financialization. SS also presents a significantly negative relationship with Fin. This implies that rigorous internal management can prevent companies from investing in high-risk financial assets. However, LnAge is positively correlated with financialization, thereby indicating that old non-financial companies might need to maintain a higher proportion of financial assets to ensure liquidity and prevent profits from being compromised by the financial industry.

### 4.2 Parallel trend test

The key logic of the DID model (1) is that the difference between the treatment group and the control group can be attributed to the ETS pilot rather than other factors. Specifically, firms within both the treatment group and the control group should exhibit the parallel financialization trends prior to the introduction of the ETS pilot, followed by significant differences after its implementation. To prove the parallel trend, we draw upon Qi et al. [6] to examine the dynamic effects of the ETS pilot on financialization of non-financial firms by constructing new independent variable, which is the product of the dummy variable Treat_i_ and the dummy variable of corresponding year (with a value of 1 in the corresponding year, and 0 otherwise). Subsequently, we build the following model (2):

$$\begin{array}{l} Fin_{i,t}=\beta_{0}+\beta_{1}pre3plus_{i,t}+\beta_{2}pre3_{i,t}+\beta_{3}pre2_{i,t}+\beta_{4}pre1_{i,t}+\\ \beta_{5}im_{i,t}+\beta_{6}post1_{i,t}+\beta_{7}post2_{i,t}+\beta_{8}post3_{i,t}+\beta_{9}post3plus_{i,t}+\\ \beta_{10}Controls_{i,t}+\mu_{j}+\gamma_{c}+\theta_{t}+\varepsilon_{i,t,j,c} \end{array}$$ (2)

where the subscript i and t mean company i and year t, respectively. Fin_i,t_ represents degree of financialization, which is measured by the ratio of financial assets to total assets, of company i in year t. pre3plus_i,t_, pre3_i,t_, pre2_i,t_, pre1_i,t_, im_i,t_, post1_i,t_, post2_i,t_, post3_i,t_, and post3plus_i,t_ are the products of the dummy variable Treat_i_ and the dummy variable of corresponding year. All other variables in model (2) remain consistent with those in model (1).

Table 3 shows the results of parallel trend test. Column (2) adds control variables to column (1). We find that the coefficients prior to ETS pilot are statistically insignificant, thereby confirming the presence of a parallel financialization trend between treatment group and control group. After the implementation of the pilot, the results signify that carbon trading policy curbs the degree of financialization of treatment group enterprises. Furthermore, this inhibiting effect remains significant more than three years after the pilot. Hence, the carbon trading policy effectively and persistently diminishes the propensity of Chinese non-financial companies to invest in financial assets.

**Table 3:** Pilot carbon trading policy and corporate financialization: Parallel trend test.

	(1)	(2)
Variable	Fin	Fin
**Pre3+**	-0.003	-0.002
	(0.004)	(0.004)
**Pre3**	-0.007	-0.005
	(0.006)	(0.006)
**Pre2**	-0.007	-0.006
	(0.005)	(0.005)
**Pre1**	-0.008	-0.007
	(0.005)	(0.005)
**Implementation**	-0.009*	-0.008
	(0.005)	(0.005)
**Post1**	-0.010*	-0.009*
	(0.005)	(0.005)
**Post2**	-0.011**	-0.010**
	(0.005)	(0.005)
**Post3**	-0.009*	-0.008*
	(0.005)	(0.005)
**Post3+**	-0.006*	-0.005*
	(0.003)	(0.003)
**Controls**	No	Yes
**Year fixed effect**	Yes	Yes
**Industry fixed effect**	Yes	Yes
**City fixed effect**	Yes	Yes
**Adj.R** ^**2**^	0.138	0.154
**Observations**	28,600	28,600

Note. This table shows the parallel trend test on the impact of pilot carbon trading policy on corporate financialization. Column (2) adds control variables to the column (1). Fin represents degree of corporate financialization, where a higher value indicates that companies are more inclined to invest in financial assets. Pre3+, Pre3, Pre2, Pre1, Implementation, Post1, Post2, Post3, Post3+ are the interaction item of the dummy variable Treat and the dummy variable of the corresponding year. Control variables are as follows: (1) ROA is the return on assets, which equals to net profits divided by total assets. (2) LnSize represents the natural logarithm of total assets. (3) Lev represents the ratio of total debt to total assets. (4) LnAge represents the natural logarithm of company age. (5) SS is the number of the supervisory board. (6) Rid represents the ratio of the number of independent directors to the total number of board directors. (7) Growth represents the growth rate of the company’s main business. Cluster-robust standard errors are in parentheses. *, **, and *** indicate significance at 10%, 5%, and 1% levels, respectively.

### 4.3 Placebo test

To assess the possibility of result bias, we conduct a placebo test by randomly selecting 15% of the 3,367 companies in full sample as the dummy treatment group, while the remaining firms are assigned as the control group. Then, we perform the random sampling with replacement three times to generate Dummy treat_1_, Dummy treat_2_, and Dummy treat_3_ to replace the variable Treat_i_ in model (1), and repeat the regression. If the estimated coefficients of Dummy treat_1_, Dummy treat_2_, and Dummy treat_3_ remain statistically significant, it suggests that our initial estimation result might be influenced by bias, indicating that changes in financialization could be affected by other policy changes or random factors, and vice versa.

Table 4 presents the results of the baseline regression and the placebo test with the dependent variable Fin. Column (1), (2), (3), and (4) show the results of Treat * Post, Dummy treat_1_* Post, Dummy treat_2_* Post, and Dummy treat_3_* Post, respectively. We find that none of the Dummy treat variables exhibit statistical significance in Column (2), (3), and (4). Consequently, the decreasing of the financialization of non-financial firms is attributed to the carbon trading policy.

**Table 4:** Pilot carbon trading policy and corporate financialization: Placebo test.

	(1)	(2)	(3)	(4)
Variable	Fin	Fin	Fin	Fin
Treat * Post	-0.005**			
	(0.002)			
**Dummy treat**_**1**_ * Post		0.001		
		(0.002)		
**Dummy treat**_**2**_ * Post			0.002	
			(0.002)	
**Dummy treat**_**3**_ * Post				-0.001
				(0.002)
**ROA**	-0.003	-0.004	-0.004	-0.004
	(0.007)	(0.008)	(0.008)	(0.008)
**LnSize**	-0.001**	-0.001**	-0.001**	-0.001**
	(0.000)	(0.000)	(0.000)	(0.000)
**Lev**	-0.042***	-0.042***	-0.042***	-0.042***
	(0.003)	(0.003)	(0.003)	(0.003)
**LnAge**	0.024***	0.024***	0.024***	0.024***
	(0.001)	(0.001)	(0.001)	(0.001)
**SS**	-0.001**	-0.001**	-0.001**	-0.001**
	(0.000)	(0.000)	(0.000)	(0.000)
**Rid**	0.003	0.003	0.003	0.003
	(0.005)	(0.005)	(0.005)	(0.005)
**Growth**	-0.001	-0.001	-0.001	-0.001
	(0.001)	(0.001)	(0.001)	(0.001)
**Year fixed effect**	Yes	Yes	Yes	Yes
**Industry fixed effect**	Yes	Yes	Yes	Yes
**City fixed effect**	Yes	Yes	Yes	Yes
**Adj.R** ^**2**^	0.154	0.154	0.154	0.154
**Observations**	28,600	28,600	28,600	28,600

Note. This table shows the placebo test on impact of pilot carbon trading policy on corporate financialization. Column (1) shows the results of baseline regression, which is the same as Column (2) in Table 2. Column (2), (3) and (4) show the results of Dummy treat_1_, Dummy treat_2_ and Dummy treat_3_, respectively. Fin represents degree of corporate financialization, where a higher value indicates that companies are more inclined to invest in financial assets. Treat is a dummy variable, which equals 1 if the firm is in the industries actually covered by the ETS pilots area, and 0 otherwise. Dummy treat is a dummy variable, which equals 1 if companies belong to the experimental group obtained by random sampling, and 0 otherwise. Post is a dummy variable that equals 1 if the year is greater than or equal to 2014, which is the starting year of trading in all ETS pilots, and 0 otherwise. Control variables are as follows: (1) ROA is the return on assets, which equals to net profits divided by total assets. (2) LnSize represents the natural logarithm of total assets. (3) Lev represents the ratio of total debt to total assets. (4) LnAge represents the natural logarithm of company age. (5) SS is the number of the supervisory board. (6) Rid represents the ratio of the number of independent directors to the total number of board directors. (7) Growth represents the growth rate of the company’s main business. Cluster-robust standard errors are in parentheses. *, **, and *** indicate significance at 10%, 5%, and 1% levels, respectively.

### 4.4 PSM-DID estimate

We also extend the DID model to incorporate propensity score matching (PSM) method in order to enhance the reliability of the research results. The control group continues to consist of firms not covered by the ETS pilot. However, by using PSM based on companies’ characteristics, we are able to identify a more suitable control group compared to the process of random selection [43].

According to Qi et al. [6], we use nearest-neighbour matching to estimate PSM-DID model. The results of the PSM balance test are shown in S2 Table. To ensure a sufficient number of observations, we match each company within the treatment group with three companies from control group. Table 5 shows the estimation results of PSM-DID. Column (2) adds control variables to the column (1). From the results, we find that the results are consistent with the baseline regression results, which proves that our research results are reliable.

**Table 5:** Pilot carbon trading policy and corporate financialization: PSM-DID.

	(1)	(2)
Variable	Fin	Fin
Treat * Post	-0.005**	-0.006**
	(0.002)	(0.002)
**ROA**		0.003
		(0.004)
**LnSize**		-0.004***
		(0.001)
**Lev**		-0.004***
		(0.001)
**LnAge**		0.020***
		(0.002)
**SS**		-0.000
		(0.000)
**Rid**		0.006
		(0.007)
**Growth**		-0.000
		(0.000)
**Year fixed effect**	Yes	Yes
**Industry fixed effect**	Yes	Yes
**City fixed effect**	Yes	Yes
**Adj.R** ^**2**^	0.126	0.137
**Observations**	13,519	13,519

Note. This table shows the impact of pilot carbon trading policy on corporate financialization by PSM-DID. Column (2) adds control variables to the column (1). Fin represents degree of corporate financialization, where a higher value indicates that companies are more inclined to invest in financial assets. Treat is a dummy variable, which equals 1 if the firm is in the industries actually covered by the ETS pilots area, and 0 otherwise. Post is a dummy variable that equals 1 if the year is greater than or equal to 2014, which is the starting year of trading in all ETS pilots, and 0 otherwise. Control variables are as follows: (1) ROA is the return on assets, which equals to net profits divided by total assets. (2) LnSize represents the natural logarithm of total assets. (3) Lev represents the ratio of total debt to total assets. (4) LnAge represents the natural logarithm of company age. (5) SS is the number of the supervisory board. (6) Rid represents the ratio of the number of independent directors to the total number of board directors. (7) Growth represents the growth rate of the company’s main business. Cluster-robust standard errors are in parentheses. *, **, and *** indicate significance at 10%, 5%, and 1% levels, respectively.

## 5. Influence channels of carbon trading policy

The analysis presented in Section 4 demonstrates that the carbon trading policy effectively reduces the financialization of non-financial firms in China. However, the mechanism of this inhibitory effect has not yet been explored. On the one hand, enterprises can obtain economic benefits by selling carbon emission rights. On the other hand, carbon trading policies can stimulate companies to disclose more about carbon emission information [15]. Based on the theory of information asymmetry, carbon information disclosure improves corporate transparency and reduces information asymmetry. It concurrently conveys to external investors a positive environment commitment, signaling that the company prioritizes ecological preservation. This signaling effect has the potential to attract more investors, thereby securing external financial support.

Through the above two analysis, it becomes evident that the carbon trading policy holds the potential to alleviate financing constraints. These constraints stand as one of the primary drivers behind companies’ heightened financial asset holdings. Hence, we explore whether carbon trading policies can reduce the degree of corporate financialization by decreasing corporate financing constraints through model (3) and (4).

$$SA_{i,t}=\beta_{0}+\beta_{1}Treat_{i}*Post_{t}+\beta_{2}Controls_{i,t}+\mu_{j}+\gamma_{c}+\theta_{t}+\varepsilon_{i,t,j,c}$$ (3)

$$Fin_{i,t}=\alpha_{0}+\alpha_{1}Treat_{i}*Post_{t}+\alpha_{2}SA_{i,t}+\alpha_{3}Controls_{i,t}+\mu_{j}+\gamma_{c}+\theta_{t}+\varepsilon_{i,t,j,c}$$ (4)

where model (3) is a benchmark DID model whose dependent variable SA_i,t_ is the measurement of corporate financing constraints. A higher value of SA_i,t_ indicates firms facing more pronounced financing constraints. In model (4), we add SA_i,t_ based on model (1). All other variables in model (3) and (4) are consistent with those in model (1). The basic idea of exploring the influence channel is to ascertain the estimation result of coefficient β_1_. If β_1_ in model (3) is statistically significant, it implies that carbon trading policies can indeed affect corporate financing constraints, and vice versa. If the significant β_1_ is along with the significant α1 and α2 in model (4), and α_1_ is significantly closer to 0 compared to β_1_ in model (1), then the financing constraint serves as the mechanism through which carbon trading policy affects the company financialization, and vice versa.

The regression results are shown in Table 6. All columns include control variables and fixed effects. Column (1), (2) and (3) show the results of model (1), (3) and (4), respectively. The significant negative coefficient of Treat * Post in column (2) indicates that the carbon trading policy eases the level of corporate financing constraints. The coefficient of SA in column (3) is significantly positive while the coefficient of Treat * Post is significantly negative and lower than the coefficient in column (1), suggesting that carbon trading policies curb financialization of non-financial firms by alleviating corporate financing constraints.

**Table 6:** Influence channel of pilot carbon trading policy.

	(1)	(2)	(3)
Variable	Fin	SA	Fin
**SA**			0.0320***
			(0.0020)
Treat * Post	-0.0053**	-0.0114*	-0.0049**
	(0.0021)	(0.0064)	(0.0021)
**Controls**	Yes	Yes	Yes
**Year fixed effect**	Yes	Yes	Yes
**Industry fixed effect**	Yes	Yes	Yes
**City fixed effect**	Yes	Yes	Yes
**Adj.R** ^**2**^	0.1543	0.4517	0.1622
**Observations**	28,600	28,600	28,600

Note. This table shows the influence channel of pilot carbon trading policy on corporate financialization. Column (1) shows the influence of pilot carbon trading policy on financialization. Column (2) shows the impact of pilot carbon trading policy on firm financing constraints. Column (3) show the effect of pilot carbon trading policy and firm financing constraints on corporate financialization. Fin represents degree of corporate financialization, where a higher value indicates that companies are more inclined to invest in financial assets. SA shows the degree of corporate financing constraints. Treat is a dummy variable, which equals 1 if the firm is in the industries actually covered by the ETS pilots area, and 0 otherwise. Post is a dummy variable that equals 1 if the year is greater than or equal to 2014, which is the starting year of trading in all ETS pilots, and 0 otherwise. Control variables are as follows: (1) ROA is the return on assets, which equals to net profits divided by total assets. (2) LnSize represents the natural logarithm of total assets. (3) Lev represents the ratio of total debt to total assets. (4) LnAge represents the natural logarithm of company age. (5) SS is the number of the supervisory board. (6) Rid represents the ratio of the number of independent directors to the total number of board directors. (7) Growth represents the growth rate of the company’s main business. Cluster-robust standard errors are in parentheses. *, **, and *** indicate significance at 10%, 5%, and 1% levels, respectively.

## 6. Heterogeneity analysis

Since we have found that carbon trading policy reduces the financialization of non-financial companies by easing financing constraints in the previous sections, the question arises: Do carbon trading policies have the same impact on companies with different characteristics? To address this issue, we explore the influence of heterogeneity on the inhibitory effect of carbon trading policy. Specifically, we analyze how differences in ownership structure, geographical locations, and industry characteristics can potentially influence the inhibitory effect of carbon trading policy on enterprises.

### 6.1 Ownership heterogeneity

The ownership structure of companies has a discernible impact on their degree of financialization [41]. Consequently, we explore how ownership heterogeneity influence the inhibitory effect of carbon trading policy.

We divide the full sample into state-owned enterprises (SOE) and non-state-owned enterprises (non-SOE), including 11,194 observations and 17,406 observations, respectively. We then re-estimate the regression of model (1). Table 7 shows the results of ownership heterogeneity. The results of SOE and non-SOE are presented in column (1) and (2), respectively. From the results, the carbon trading policy only decrease the financialization of non-state non-financial enterprises.

**Table 7:** Pilot carbon trading policy and corporate financialization: Ownership heterogeneity.

	(1)	(2)
	SOE	Non-SOE
Variable	Fin	Fin
Treat * Post	-0.001	-0.007**
	(0.003)	(0.003)
**ROA**	-0.044***	0.004
	(0.013)	(0.009)
**LnSize**	-0.002***	0.001
	(0.001)	(0.001)
**Lev**	-0.050***	-0.043***
	(0.004)	(0.004)
**LnAge**	0.022***	0.019***
	(0.003)	(0.002)
**SS**	0.001*	-0.001***
	(0.000)	(0.000)
**Rid**	0.012	-0.001
	(0.008)	(0.006)
**Growth**	-0.002	-0.000
	(0.001)	(0.001)
**Year fixed effect**	Yes	Yes
**Industry fixed effect**	Yes	Yes
**City fixed effect**	Yes	Yes
**Adj.R** ^**2**^	0.340	0.139
**Observations**	11,194	17,406

Note. This table shows the ownership heterogeneity of pilot carbon trading policy on corporate financialization. Column (1) and (2) show the results of state-owned enterprise and non-state-owned enterprise, respectively. Fin represents degree of corporate financialization, where a higher value indicates that companies are more inclined to invest in financial assets. Treat is a dummy variable, which equals 1 if the firm is in the industries actually covered by the ETS pilots area, and 0 otherwise. Post is a dummy variable that equals 1 if the year is greater than or equal to 2014, which is the starting year of trading in all ETS pilots, and 0 otherwise. Control variables are as follows: (1) ROA is the return on assets, which equals to net profits divided by total assets. (2) LnSize represents the natural logarithm of total assets. (3) Lev represents the ratio of total debt to total assets. (4) LnAge represents the natural logarithm of company age. (5) SS is the number of the supervisory board. (6) Rid represents the ratio of the number of independent directors to the total number of board directors. (7) Growth represents the growth rate of the company’s main business. Cluster-robust standard errors are in parentheses. *, **, and *** indicate significance at 10%, 5%, and 1% levels, respectively.

The different financial asset holding strategies of state-owned enterprises and non-state-owned enterprises in response to carbon trading policies may be attributed to several reasons. First, the innate political connection between state-owned enterprises and the government makes the latter become a guarantor for the former. In times of financial distress for non-financial state-owned enterprises resulting from reduced profits, the government subsidies can help alleviate this situation. Moreover, the connection between the government and the bank reduces the difficulty of financing for state-owned enterprises. Second, preventing major financial risks is one of the main strategies of the Chinese government. As a stable of regional economic development and employment, state-owned enterprises may be regulated by government to refrain from holding substantial high-risk financial assets. Both factors contribute to state-owned enterprises maintaining a lower level of financialization before the carbon trading policies, rendering them less sensitive to the inhibitory effects of carbon trading policies. Conversely, non-state-owned enterprises tend to hold more financial assets in order to survive in the context of the downturn in the entities economy, leading them more susceptible to the pronounced influence of carbon trading policies.

### 6.2 Regional heterogeneity

China’s vastness encompasses a spectrum of regional economic development levels. Differences in regional macroeconomic factors can significantly affect a company’s operating environment, thereby changing their financial investment decisions [44]. Hence, we verify whether regional heterogeneity plays a role in shaping the effectiveness of carbon trading policies.

Based on the location of the company, we divide the total sample into two sub-samples: companies located in the eastern region, and those in the central and western regions. We then re-estimate model (1) on each of the sub-samples. Table 8 shows the results of regional heterogeneity. Column (1) and (2) show the results for firms in eastern region firms and those in central and western regions, respectively. We find that the carbon trading policy only decrease the financialization of non-financial firms located in eastern region.

**Table 8:** Pilot carbon trading policy and corporate financialization: Regional heterogeneity.

	(1)	(2)
	Eastern region	Central and western regions
Variable	Fin	Fin
Treat * Post	-0.004*	-0.007
	(0.002)	(0.006)
**ROA**	-0.017*	0.022**
	(0.010)	(0.011)
**LnSize**	0.002***	-0.007***
	(0.001)	(0.001)
**Lev**	-0.060***	-0.003
	(0.004)	(0.004)
**LnAge**	0.025***	0.022***
	(0.002)	(0.003)
**SS**	-0.001***	0.000
	(0.000)	(0.000)
**Rid**	0.008	-0.009
	(0.006)	(0.008)
**Growth**	-0.002*	0.001
	(0.001)	(0.001)
**Year fixed effect**	Yes	Yes
**Industry fixed effect**	Yes	Yes
**City fixed effect**	Yes	Yes
**Adj.R** ^**2**^	0.162	0.154
**Observations**	19,773	8,827

Note. This table shows the regional heterogeneity of pilot carbon trading policy on corporate financialization. Column (1) and (2) show the results of eastern region and central and western regions, respectively. Fin represents degree of corporate financialization, where a higher value indicates that companies are more inclined to invest in financial assets. Treat is a dummy variable, which equals 1 if the firm is in the industries actually covered by the ETS pilots area, and 0 otherwise. Post is a dummy variable that equals 1 if the year is greater than or equal to 2014, which is the starting year of trading in all ETS pilots, and 0 otherwise. Control variables are as follows: (1) ROA is the return on assets, which equals to net profits divided by total assets. (2) LnSize represents the natural logarithm of total assets. (3) Lev represents the ratio of total debt to total assets. (4) LnAge represents the natural logarithm of company age. (5) SS is the number of the supervisory board. (6) Rid represents the ratio of the number of independent directors to the total number of board directors. (7) Growth represents the growth rate of the company’s main business. Cluster-robust standard errors are in parentheses. *, **, and *** indicate significance at 10%, 5%, and 1% levels, respectively.

The reasons for this situation may be that the rapid economic development in the eastern China has led to a high-density distribution of firms, resulting in heightened competition pressures and increased difficulties in financing within this region. Consequently, non-financial companies in the eastern region have to hold more financial assets under the dual pressure of competition and market shrinkage before the carbon trading policy. This heightened financialization makes these companies particularly sensitive to reduced financing constraints and additional benefits brought about by the carbon trading policy. However, for central and western regions’ companies, on the one hand, the low density of firms presence lead to less competition pressure. On the other hand, the Chinese government has extended substantial support policies to these regions in order to achieve the strategic goal of common prosperity. As a result, enterprises situated in central and western regions encounter relatively diminished financing challenges due to the government’s assistance. Hence, low competition and relatively sufficient funds make companies in central and western regions less responsive to direct or indirect benefit from carbon trading policies.

### 6.3 Industry competition heterogeneity

In this section, we verify the impact of industry competition on the effect of carbon trading policies. According to the view of Rhoades [45], Tingvall and Poldahl [46], and Kvålseth [47], we use Herfindahl index to measure the level of industry competition. We compute the average Herfindahl index for each industry from the initiation of the sample until the implementation of ETS pilots trading based on total assets. We then classify the industries whose average Herfindahl index below the median as highly competitive industries, and others as industries with less competition. Referring to the 2012 edition of the China Securities Regulatory Commission industry classification, we divide the total sample into two sub-samples of companies in highly competitive industries and firms in non-high competition industries. Table 9 shows the results of industry competition heterogeneity. Column (1) and (2) show the results of companies operating within highly competitive industries and firms with lower competition levels, respectively.

**Table 9:** Pilot carbon trading policy and corporate financialization: Industry competition heterogeneity.

	(1)	(2)
	High competition	Low competition
Variable	Fin	Fin
Treat * Post	-0.004*	-0.017
	(0.002)	(0.010)
**ROA**	0.002	-0.030
	(0.008)	(0.022)
**LnSize**	-0.002***	0.002
	(0.000)	(0.002)
**Lev**	-0.038***	-0.074***
	(0.003)	(0.010)
**LnAge**	0.020***	0.056***
	(0.001)	(0.006)
**SS**	-0.000*	-0.001*
	(0.000)	(0.001)
**Rid**	0.001	0.008
	(0.005)	(0.019)
**Growth**	-0.003**	0.002
	(0.001)	(0.002)
**Year fixed effect**	Yes	Yes
**Industry fixed effect**	Yes	Yes
**City fixed effect**	Yes	Yes
**Adj.R** ^**2**^	0.139	0.255
**Observations**	24,911	3,617

Note. This table shows the industry heterogeneity of pilot carbon trading policy on corporate financialization. Column (1) and (2) show the results of high competition industry and low competition industry, respectively. Fin represents degree of corporate financialization, where a higher value indicates that companies are more inclined to invest in financial assets. Treat is a dummy variable, which equals 1 if the firm is in the industries actually covered by the ETS pilots area, and 0 otherwise. Post is a dummy variable that equals 1 if the year is greater than or equal to 2014, which is the starting year of trading in all ETS pilots, and 0 otherwise. Control variables are as follows: (1) ROA is the return on assets, which equals to net profits divided by total assets. (2) LnSize represents the natural logarithm of total assets. (3) Lev represents the ratio of total debt to total assets. (4) LnAge represents the natural logarithm of company age. (5) SS is the number of the supervisory board. (6) Rid represents the ratio of the number of independent directors to the total number of board directors. (7) Growth represents the growth rate of the company’s main business. Cluster-robust standard errors are in parentheses. *, **, and *** indicate significance at 10%, 5%, and 1% levels, respectively.

Through the results, we find that the carbon trading policy only decrease the financialization of non-financial firms within highly competitive industries. The plausible explanation is that in industries with low market competition, most companies have a certain degree of monopoly, potentially affording them sufficient control over market dynamics. Consequently, the pressures pertaining to financing and operations are relatively low in such contexts. In addition, industries characterized by low market competition are often occupied by state-owned enterprises, which tend to enjoy close affiliations with the government bodies. This intricate relationship leads them less responsive to the additional advantages caused by carbon trading policy. Conversely, for companies in industries with a high degree of market competition, their investment decisions appear to exhibit a heightened level of sensitivity to the supplementary benefits stemming from carbon trading policy.

## 7. Conclusion and discussion

Reducing carbon emission plays an important role in alleviating global warming. Simultaneously, the intensification of financialization trends of non-financial enterprises has gained attention. The harm of corporate financialization has gradually emerged. Nevertheless, existing research has neglected the potential contradictions between environmental regulations and financialization, resulting in an unclear relationship between carbon trading policy and financialization of non-financial firms.

To address this gap, we employ China emission trading scheme pilots as a quasi-natural experiment and use the difference-in-differences model to study the impact of carbon trading policy on financialization of non-financial companies. In doing so, we effectively identify the causal relationship between carbon trading policies and corporate financialization, and eliminate the influence of non-result related factors. We not only expand the research on the impact of carbon trading policy on micro-company, but also investigate the influence factor of corporate financialization. Moreover, our research results provide a policy reference for reducing the corporate financialization in emerging markets under low-carbon transition.

According to our results, carbon trading policy can effectively restrain the financialization of non-financial enterprises through reducing firms’ financing constraints. In parallel trend test, we find that the inhibitory effect of ETS pilot on corporate financialization consistently remains significant. Our findings show robust validity even when (1) extending the DID model to a PSM-DID model to get a more appropriate control group, and (2) using the placebo test to verify possible results bias. Finally, we conduct a cross-sectional test in terms of company ownership, company location, and industry competition, and find that carbon trading policy exhibits a more significant inhibitory effect on the financialization of (1) non-state-owned enterprise, (2) companies in eastern region, and (3) firms operating within highly competitive industries. In summary, emerging economies can achieve a win-win situation by implementing carbon trading policies that foster both low-carbon transition and stable economic development.

Drawing from our results, the implementation of the carbon trading policy in China has yielded favorable outcomes. Hence, it is imperative to propagate it from its initial pilot phase to a nationwide scope. However, the amplification of this policy demands meticulous attention to regional heterogeneity. Specifically, our findings underscore that the carbon trading policy’s impact on curbing the financialization tendencies of non-financial enterprises remains limited in the western regions. This nuance necessitates that the government should formulate complementary incentive frameworks to stimulate active engagement of regional enterprises in carbon trading initiatives during policy propagation in the western areas. Moreover, our research also illuminates that the reduction of financialization among state-owned non-financial enterprises is not significant after carbon trading policy. In such instances, the government’s direct intervention via administrative directives emerges as a viable strategy to curtail the extent of financialization within state-owned non-financial enterprises.

Our study has solely delved into the impact of carbon trading policies on the financialization of non-financial enterprises. However, within emerging markets, obligatory carbon reduction policies based on administrative mandates still prevail. In the future, we intend to delve deeper into the effects of mandatory carbon reduction policies on corporate financialization, aiming to compare the applicability of these differing policies within emerging markets.

## Supporting information

S1 Table Variable definitions. (DOCX)

S2 Table Test of covariate balancing in nearest-neighbour matching method. (DOCX)

S1 Data (DTA)
