# Supplementary material for: How do carbon emissions trading impact the financialization of non-financial companies? Evidence from a quasi-natural experiment in China
Source: PLoS One. 2023 Dec 27;18(12):e0296277. doi: 10.1371/journal.pone.0296277 (PMC10752544; doi:10.1371/journal.pone.0296277)
Supplement: S1 Table — (DOCX) [file pone.0296277.s001.docx]

**Supporting information**

**S1 Table. Variable definitions**

| **Variable** | **Definitions** |
| --- | --- |
| **Fin** | The degree of corporate financialization measured by the ratio of financial assets to total assets |
| **Treat** | Dummy variable, Treat equals 1 if the firm is in the industries actually covered by the ETS pilots area, and 0 otherwise |
| **Post** | Dummy variable, Post equals 1 if the year is greater than or equal to 2014, which is the starting year of trading in all ETS pilots, and 0 otherwise |
| **ROA** | Return on assets, which equals to net profits divided by total assets |
| **LnSize** | The natural logarithm of total assets |
| **Lev** | Total debt divided by total assets |
| **LnAge** | The natural logarithm of company age |
| **SS** | The number of the supervisory board |
| **Rid** | The ratio of the number of independent directors to the total number of board directors |
| **Growth** | The growth rate of the company’s main business |
