# Supplementary material for: How do carbon emissions trading impact the financialization of non-financial companies? Evidence from a quasi-natural experiment in China
Source: PLoS One. 2023 Dec 27;18(12):e0296277. doi: 10.1371/journal.pone.0296277 (PMC10752544; doi:10.1371/journal.pone.0296277)
Supplement: S2 Table — (DOCX) [file pone.0296277.s002.docx]

**Supporting information**

**S2 Table. Test of covariate balancing in nearest-neighbour matching method**

|  |  | **Mean** | |  |  | **T-test** | |
| --- | --- | --- | --- | --- | --- | --- | --- |
| **Variable** |  | **Treated** | **Control** | **%bias** | **%reduct**  **bias** | **t** | ***p* > t** |
| **ROA** | Unmatched | 0.038 | 0.035 | 3.9 | 68.3 | 2.08 | 0.037 |
|  | matched | 0.038 | 0.037 | 1.2 |  | 0.52 | 0.605 |
| **LnSize** | Unmatched | 22.290 | 22.021 | 19.9 | 94.0 | 11.84 | 0.000 |
|  | matched | 22.279 | 22.295 | -1.2 |  | -0.48 | 0.634 |
| **Lev** | Unmatched | 0.422 | 0.428 | -3.0 | -20.3 | -1.62 | 0.106 |
|  | matched | 0.422 | 0.429 | -3.6 |  | -1.48 | 0.139 |
| **LnAge** | Unmatched | 2.696 | 2.761 | -16.2 | 97.8 | -9.28 | 0.000 |
|  | matched | 2.701 | 2.699 | 0.4 |  | 0.14 | 0.888 |
| **SS** | Unmatched | 4.383 | 4.473 | -4.3 | 83.7 | -2.30 | 0.021 |
|  | matched | 4.388 | 4.402 | -0.7 |  | -0.30 | 0.766 |
| **Rid** | Unmatched | 0.394 | 0.387 | 6.3 | 92.9 | 3.47 | 0.001 |
|  | matched | 0.393 | 0.394 | -0.4 |  | -0.19 | 0.851 |
| **Growth** | Unmatched | 0.177 | 0.172 | 1.1 | -18.4 | 0.60 | 0.547 |
|  | matched | 0.177 | 0.171 | 1.3 |  | 0.56 | 0.575 |
